# Trends in Unprotected Left Main Percutaneous Coronary Intervention and Clinical Outcomes

**DOI:** 10.1001/jamanetworkopen.2025.60422

**Published:** 2026-02-23

**Authors:** Nobuhiro Ikemura, Yuichiro Mori, Paul S. Chan, David J. Cohen, Kyohei Yamaji, Tetsuya Amano, Ken Kozuma, John A. Spertus, Shun Kohsaka

**Affiliations:** 1 Healthcare Institute for Innovations in Quality, University of Missouri–Kansas City; 2 Saint Luke’s Mid America Heart Institute, Kansas City, Missouri; 3 Keio University School of Medicine, Tokyo, Japan; 4 Graduate School of Medicine, Kyoto University, Kyoto, Japan; 5 Cardiovascular Research Foundation, New York, New York; 6 St Francis Hospital, Roslyn, New York; 7 Department of Cardiovascular Medicine, Kyoto University Graduate School of Medicine, Kyoto, Japan; 8 Department of Cardiology, Aichi Medical University, Nagakute, Japan; 9 Teikyo University Hospital, Tokyo, Japan

## Abstract

**Importance:**

Unprotected left main percutaneous coronary intervention (LM PCI) is recognized as a reasonable strategy for patients who have coronary artery disease with low anatomic complexity. However, recent trends in its procedural volume, patient characteristics, and procedural techniques and their association with clinical outcomes have not been well described, making it important to confirm the safety of LM PCI in contemporary clinical practice.

**Objectives:**

To examine temporal trends in the use and in-hospital outcomes of unprotected LM PCI in Japan.

**Design, Setting, and Participants:**

This retrospective cohort study used prospectively collected data from the nationwide, multicenter Japanese Percutaneous Coronary Intervention registry including approximately 1100 PCI-capable institutions across Japan. Data were restricted to the period after the registry’s 2019 update to ensure consistency in definitions. Temporal trends in PCI procedures were analyzed from January 2019 through December 2023. Patients with prior coronary artery bypass grafting, recent cardiogenic shock, cardiac arrest within 24 hours of treatment, restenosis, or balloon angioplasty alone were excluded.

**Exposures:**

Calendar year of PCI (2019-2023).

**Main Outcomes and Measures:**

The primary outcome was in-hospital mortality. Multivariable logistic regression was used to assess temporal trends and whether differences were explained by baseline or procedural characteristics.

**Results:**

Among 851 468 included procedures (mean [SD] patient age, 74.1 [10.2] years; 78.1% men), unprotected LM PCI accounted for 9490 of 182 739 PCI procedures in 2019 (5.2%) and 8583 of 164 332 in 2023 (5.2%). From 2019 to 2023, mean (SD) patient age increased from 73.4 (10.1) to 74.6 (10.1) years, and patients were more likely to have heart failure (increasing from 1676 of 9058 cases in 2019 [18.5%] to 1704 of 8200 [20.8%] in 2023). Unadjusted in-hospital mortality increased from 148 cases in 2019 (1.6%) to 157 in 2023 (1.9%), but this was not significant after adjusting for patient characteristics (adjusted odds ratio for 2023 vs 2019: 1.08; 95% CI, 0.85-1.37). From 2019 to 2023, further adjustment for procedural factors, including increased use of radial access (from 5789 cases [63.9%] to 5985 cases [73.0%]) and mechanical circulatory support (from 828 cases [9.1%] to 941 cases [11.5%]), did not alter the association between year and mortality.

**Conclusions and Relevance:**

In a nationwide Japanese PCI registry, unprotected LM PCI consistently accounted for 5% of PCI procedures. Although unadjusted in-hospital mortality rose modestly over time, the findings indicate this was largely explained by increasing patient complexity, with risk-adjusted outcomes remaining stable, supporting the continued role of LM PCI as a safe revascularization option in contemporary practice.

## Key Points

**Question:**

What are recent temporal trends in the use and in-hospital outcomes of unprotected left main percutaneous coronary intervention (LM PCI) in Japan?

**Findings:**

In this nationwide cohort study of 851 468 PCI procedures (2019-2023), unprotected LM PCI consistently represented about 5% of all cases. Mean patient age increased and patients had more comorbidities over time, while unadjusted in-hospital mortality rose slightly (from 1.6% in 2019 to 1.9% in 2023); however, after adjustment for patient and procedural characteristics, mortality rates remained stable.

**Meaning:**

The findings of this study suggest that LM PCI has been increasingly adopted in higher-risk patients in Japan while maintaining stable short-term outcomes, supporting its continued role in contemporary revascularization practice.

## Teaser

This cohort study investigates temporal trends in the use of unprotected left main percutaneous coronary intervention and its in-hospital outcomes from 2019 to 2023 in a national registry of patients in Japan.

## Introduction

Historically, left main (LM) coronary artery disease has been managed primarily with coronary artery bypass grafting (CABG) as the standard of care.^1,2^ However, some patients have high surgical risk, absolute or relative contraindications to surgery, or strong preferences for less invasive treatment, making percutaneous coronary intervention (PCI) a necessary alternative.^3^ Randomized clinical trials and subsequent meta-analyses have demonstrated that PCI for unprotected LM coronary artery disease achieves 5-year clinical outcomes comparable to CABG in patients with low anatomic complexity.^4,5^ Accordingly, both European and Japanese guidelines endorse PCI for unprotected LM coronary artery disease as an equivalent alternative to CABG (class I recommendation) in patients with low anatomic complexity.^1,6^

Practice patterns in unprotected LM PCI, along with changes in patient characteristics and clinical outcomes, remain largely unexplored. Previous reports from the US and the UK describe a modest increase in LM PCI volumes from 2009 to 2017^7,8^; however, more recent trends have not been well documented. Moreover, advances in PCI procedural techniques, such as the use of radial access,^9^ mechanical circulatory support (MCS),^10^ and intravascular imaging,^11^ may have reshaped procedural strategies. Understanding the impact of temporal changes in patient and procedural characteristics on the use and outcomes of LM PCI is important to confirm the safety of this approach in contemporary care.

To address this gap in knowledge, we leveraged data from the Japanese Percutaneous Coronary Intervention (J-PCI) registry to examine the contemporary trends in the use of unprotected LM PCI and changes in in-hospital outcomes. Given that LM revascularization remains a critical but underrepresented topic in randomized clinical trials,^12,13^ the J-PCI registry provides a unique opportunity to assess clinical practice patterns and identify opportunities to improve the quality of care in this complex patient population.

## Methods

The study protocol for the J-PCI registry was approved by the institutional review board committee at the Network for Promotion of Clinical Studies, a nonprofit organization affiliated with Osaka University Graduate School of Medicine, Osaka, Japan.^14,15^ The current cohort study adhered to the ethical principles outlined in the Declaration of Helsinki.^16^ Given the study’s retrospective and observational design, the requirement for written informed consent was waived by the institutional review board of the Japan Conference of Clinical Research, Tokyo. This report follows the Strengthening the Reporting of Observational Studies in Epidemiology (STROBE) reporting guideline.^17^

### Data Source

The J-PCI registry is a prospective, nationwide, multicenter registry in Japan, established by the Japanese Association of Cardiovascular Intervention and Therapeutics, a professional academic society dedicated to cardiac interventions.^14,15^ Designed to systematically collect data on clinical characteristics and in-hospital outcomes of patients undergoing PCI, the J-PCI registry covers approximately 90% of all Japanese PCI procedures.^18^ Since January 2013, the J-PCI registry has been integrated into Japan’s National Clinical Database, a nationwide, prospective, internet-based registry linked to board certification.^14,15^ Each participating hospital designates a data manager responsible for collecting and inputting PCI data into the centralized database.^14,15^ Approximately 1100 institutions record more than 250 000 PCI cases annually.^15^ To ensure data accuracy and reliability, Cardiovascular Intervention and Therapeutics conducts an annual meeting for data managers and performs random audits at 20 institutions per year.^14,15^ These audits assess both the completeness of case registration and the accuracy of key clinical variables by comparing source documents with registry entries and have verified that both case inclusion and key clinical variables are recorded with high accuracy, without evidence of systematic errors.^14,15^

### Study Population

We identified procedures performed at institutions participating in the J-PCI registry from January 2019 through December 2023. We limited the analysis to this period because the registry underwent a major update in 2019, ensuring consistent data collection and outcome definitions necessary for reliable trend assessment. Procedures were excluded if patients had a history of CABG, regardless of graft patency, as prior bypass surgery provides an alternative revascularization route and alters the native coronary anatomy, making interpretation of LM PCI outcomes less reliable. Procedures were also excluded if patients experienced cardiogenic shock or cardiac arrest within 24 hours of treatment, so that the study could focus on noncritical practice patterns. Similarly, interventions for restenosis lesions were excluded. Lastly, patients undergoing balloon angioplasty alone were excluded, as these cases may represent stabilizing treatment before CABG. In addition, procedures with missing data for any of the exclusion criteria were excluded.

### Key Variables and Outcomes

The independent variable of calendar year was modeled as a categorical variable, with 2019 as the reference. Adjusted odds ratios (AORs) were estimated for each subsequent year (2020-2023) compared with 2019. The primary outcome was in-hospital mortality. Secondary outcomes included in-hospital mortality due to primary cardiovascular cause^19^ and the composite outcome of in-hospital mortality and procedure-related complications, including cardiogenic shock or acute heart failure requiring mechanical support or inotropes, bleeding events requiring blood transfusion, cardiac tamponade, stent thrombosis based on the Academic Research Consortium definition, and emergency surgery. Given the well-established prognostic differences between patients with acute coronary syndrome (ACS) and those with non-ACS, an additional analysis was conducted to compare trends in both clinical situations.

### Statistical Analysis

Patient characteristics, LM lesion information, and procedural details were summarized by enrollment year. The data are presented as mean (SD) for continuous data and as frequency (percentage) for categorical data. Cochran-Armitage analyses were used to describe unadjusted temporal trends.

To examine temporal trends in the primary outcome of in-hospital mortality, a multivariable logistic regression model was used to adjust for the following variables as fixed effects in addition to calendar year: patient age (per 1-year increase), sex, presentation (ACS vs non-ACS), PCI status (elective vs nonelective), smoking, diabetes, hypertension, dyslipidemia, peripheral artery disease, chronic kidney disease (eg, estimated glomerular filtration rate <60 mL/min/1.73 m^2^), dialysis, chronic obstructive lung disease, prior history of PCI, myocardial infarction, heart failure, and a history of acute heart failure with 24 hours. To assess the extent to which differences in procedural characteristics by enrollment year accounted for differences in in-hospital mortality, we further adjusted for vascular access site (transradial vs others), use of MCS during the procedure, atherectomy device, and number of treated vessels. We included use of MCS as a covariate in our model, recognizing that it may serve as a marker of illness severity rather than a direct mediator of adverse outcomes. Although intracoronary imaging is known to improve PCI outcomes^11^ and is increasingly recommended for complex lesions in guidelines,^2^ we were unable to include it in the adjustment model because the J-PCI registry does not collect detailed information on its use, given its near-universal adoption in Japan.^20^ Results are presented as AORs with 95% CIs for in-hospital mortality, using 2019 as the reference year. Given the very low event rate of in-hospital mortality and the fact that most participating facilities experienced no LM PCI–related deaths during the study period,^21^ incorporation of facility- or operator-level random effects was not statistically feasible and could have resulted in model instability.

These analyses were repeated for the secondary composite outcome of in-hospital mortality and procedure-related complications. In addition, within the primary model described (ie, adjusted for both baseline and procedural characteristics), we tested for interactions between calendar year and presentation (ACS vs non-ACS) to assess potential differential associations. The 95% CIs and *P* values were derived using a Poisson distribution. All tests were 2-sided, with *P* < .05 indicating significance. Analyses were completed with R, version 4.4.3 (R Project for Statistical Computing).

## Results

### Study Cohort and Trends in Unprotected LM PCI

**Figure 1.**
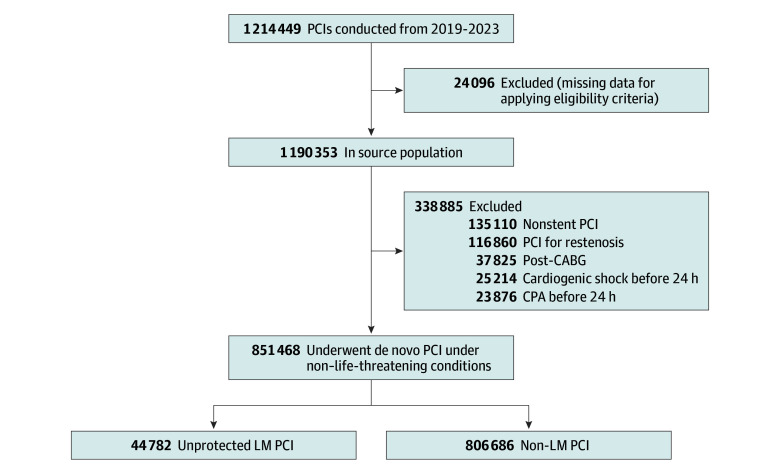
Study Flowchart CABG indicates coronary artery bypass graft; CPA, cardiopulmonary arrest; LM, left main; PCI, percutaneous coronary intervention.

Of 1 214 449 identified procedures in the J-PCI registry, 37 825 (3.1%) were excluded because patients had a history of CABG, 49 090 (4.0%) because patients experienced cardiogenic shock or cardiac arrest within 24 hours of treatment, 116 860 (9.6%) because of interventions for restenosis lesions, and 135 110 (11.1%) because patients underwent balloon angioplasty alone. An additional 24 096 (2.0%) were excluded due to missing data for eligibility criteria. The final analytic cohort included 851 468 de novo PCI procedures under non–life-threatening conditions (mean [SD] patient age, 74.1 [10.2] years; 78.1% men, 21.9% women), of which 67.0% were for non-ACS presentation and 78.6% were elective. A total of 44 782 procedures (5.3%) were for unprotected LM coronary artery disease (Figure 1).

**Figure 2.**
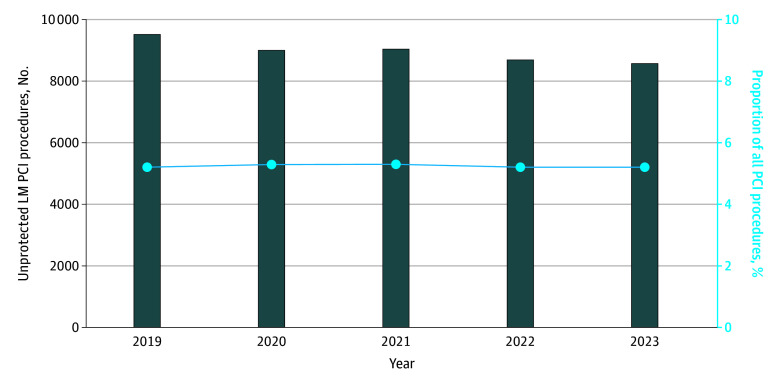
Bar Graph of Temporal Trends in Unprotected Left Main Percutaneous Coronary Intervention (LM PCI) The horizontal line indicates the proportion, as a percentage, among all PCI procedures.

Throughout the observation period, the rate of unprotected LM PCI procedures remained largely unchanged, ranging from 9490 of 182 739 procedures (5.2%) in 2019 to 8583 of 164 332 (5.2%) in 2023 (Figure 2). eTable 1 in Supplement 1 summarizes the number of participating institutions and the total number of PCI procedures performed annually during the study period. While the number of participating sites increased over time, the overall volume of PCI procedures slightly decreased. Accordingly, the proportion of unprotected LM PCI remained stable as overall PCI volumes declined.

### Trends in Patient Characteristics and Clinical Outcomes

For the outcome analysis, 2124 patients with missing data for 1 or more covariates required for the multivariable analysis (4.7%) were excluded, resulting in a final analytic cohort of 42 658 unprotected LM PCI cases. Table 1 summarizes characteristics of patients receiving unprotected LM PCI across enrollment years. The mean (SD) age increased over the 5-year study period (from 73.4 [10.1] years in 2019 to 74.6 [10.1] years in 2023), while the sex distribution remained stable. The prevalence of most cardiovascular risk factors increased, except for current smoking. The prevalence of a history of heart failure increased (1676 of the 9058 cases in 2019 [18.5%] vs 1704 of the 8200 in 2023 [20.8%]), whereas a history of prior PCI decreased. The proportion of procedures performed for ACS remained stable throughout the study period (2910 [32.1%] in 2019 vs 2690 [32.8%] in 2023). In terms of procedural characteristics, the use of radial artery access increased (5789 cases [63.9%] in 2019 vs 5985 [73.0%] in 2023), as did the use of MCS (828 [9.1%] in 2019 vs 941 [11.5%] in 2023). Furthermore, the proportion of patients who underwent LM-to–left anterior descending intervention only (ie, single-vessel LM PCI) showed an increasing trend over time (1773 [19.6%] in 2019 vs 1747 [21.3%] in 2023).

**Table 1.** Baseline Characteristics

Characteristic	Unprotected LM PCI cases, No. (%) (N = 42 658)				
	2019 (n = 9058)	2020 (n = 8625)	2021 (n = 8412)	2022 (n = 8363)	2023 (n = 8200)
Participating sites, No.	952	953	960	978	991
PCI operators, No.	3700	3715	3768	3829	3873
Age, mean (SD), y	73.4 (10.1)	73.9 (10.2)	74.2 (10.3)	74.4 (10.3)	74.6 (10.1)
Sex					
Female	1952 (21.5)	1946 (22.5)	1869 (22.2)	1821 (21.7)	1756 (21.4)
Male	7106 (78.4)	6679 (77.4)	6543 (77.8)	6542 (78.2)	6444 (78.6)
Presentation					
Non-ACS	6148 (67.9)	5888 (68.3)	5525 (65.7)	5503 (65.8)	5510 (67.2)
ACS	2910 (32.1)	2737 (31.7)	2887 (34.3)	2860 (34.2)	2690 (32.8)
Elective PCI	7250 (80.0)	6871 (79.7)	6586 (78.3)	6470 (77.4)	6370 (77.7)
Current smoker	2586 (28.5)	2419 (28.0)	2303 (27.4)	2302 (27.5)	2149 (26.2)
Comorbidities					
Diabetes	4261 (47.0)	4063 (47.1)	3901 (46.4)	3932 (47.0)	4009 (48.9)
Hypertension	7071 (78.1)	6814 (79.0)	6707 (79.7)	6657 (79.6)	6542 (79.8)
Dyslipidemia	6290 (69.4)	6117 (70.9)	5935 (70.6)	5941 (71.0)	5808 (70.8)
Peripheral artery disease	882 (9.7)	915 (10.6)	823 (9.8)	785 (9.4)	776 (9.5)
Chronic kidney disease	2237 (24.7)	2317 (26.9)	2295 (27.3)	2527 (30.2)	2500 (30.5)
Chronic lung disease	295 (3.3)	298 (3.5)	316 (3.8)	316 (3.8)	302 (3.7)
Patient receiving maintenance dialysis	607 (6.7)	600 (7.0)	545 (6.5)	527 (6.3)	523 (6.4)
Prior PCI	4471 (49.4)	4342 (50.3)	3843 (45.7)	3839 (45.9)	3869 (47.2)
Prior myocardial infarction	2022 (22.3)	1992 (23.1)	1827 (21.7)	1899 (22.7)	1864 (22.7)
Prior HF	1676 (18.5)	1736 (20.1)	1689 (20.1)	1724 (20.6)	1704 (20.8)
Acute HF within 24 h	368 (4.1)	317 (3.7)	366 (4.4)	351 (4.2)	316 (3.9)
Access site					
Femoral	2867 (31.7)	2458 (28.5)	2283 (27.1)	2038 (24.4)	1897 (23.1)
Radial	5789 (63.9)	5780 (67.0)	5745 (68.3)	5972 (71.4)	5985 (73.0)
Other	402 (4.4)	387 (4.5)	384 (4.6)	353 (4.2)	318 (3.9)
Mechanical circulatory support device					
Overall	828 (9.1)	864 (10.0)	1001 (11.9)	1006 (12.0)	941 (11.5)
IABP	790 (8.7)	807 (9.4)	930 (11.1)	914 (10.9)	850 (10.4)
ECMO	80 (0.9)	69 (0.8)	69 (0.8)	64 (0.8)	76 (0.9)
Impella	29 (0.3)	40 (0.5)	69 (0.8)	81 (1.0)	76 (0.9)
Type of device used					
Bare metal stent	34 (0.4)	12 (0.1)	13 (0.2)	22 (0.3)	15 (0.2)
Atherectomy device	700 (7.7)	642 (7.4)	773 (9.2)	760 (9.1)	620 (7.6)
Treated vessels, No.					
1	1773 (19.6)	1693 (19.6)	1696 (20.2)	1648 (19.7)	1747 (21.3)
2	4893 (54.0)	4764 (55.2)	4599 (54.7)	4688 (56.1)	4505 (54.9)
3	2335 (25.8)	2125 (24.6)	2071 (24.6)	1981 (23.7)	1904 (23.2)

Abbreviations: ACS, acute coronary syndrome; ECMO, extracorporeal membrane oxygenation; HF, heart failure; IABP, intra-aortic balloon pump; LM, left main; PCI, percutaneous coronary intervention.

Unadjusted in-hospital mortality among patients undergoing unprotected LM PCI increased slightly from 148 cases (1.6%) in 2019 to 157 (1.9%) in 2023 (*P* for trend = .03) (Table 2). For the secondary outcomes, the composite of in-hospital mortality and procedure-related complications also increased slightly from 316 cases (3.5%) in 2019 to 331 (4.0%) in 2023 (*P* for trend = .02) (Table 2). This increase was driven in part by a rise in access-site bleeding complications over time (30 cases [0.3%] in 2019 to 49 [0.6%] in 2023; *P* for trend = .02). In the subgroup analysis by ACS and non-ACS presentations, in-hospital mortality remained stable over time in both groups (eTable 2 in Supplement 1).

**Table 2.** Trends in Clinical Outcomes After Unprotected LM PCI

Outcome	Unprotected LM PCI cases, No. (%) (N = 42 658)					*P* for trend^a^
	2019 (n = 9058)	2020 (n = 8625)	2021 (n = 8412)	2022 (n = 8363)	2023 (n = 8200)	
In-hospital mortality	148 (1.6)	137 (1.6)	142 (1.7)	170 (2.0)	157 (1.9)	.03
Cardiovascular mortality	115 (1.3)	108 (1.3)	106 (1.3)	128 (1.5)	114 (1.4)	.19
Composite outcome of in-hospital mortality and procedural complications	316 (3.5)	295 (3.4)	343 (4.1)	320 (3.8)	331 (4.0)	.02
Cardiogenic shock or acute heart failure requiring mechanical support or inotropes	134 (1.5)	135 (1.6)	160 (1.9)	124 (1.5)	147 (1.8)	.20
Bleeding event requiring blood transfusion						
Overall	55 (0.6)	56 (0.6)	74 (0.9)	53 (0.6)	74 (0.9)	.047
Access site	30 (0.3)	34 (0.4)	40 (0.5)	30 (0.4)	49 (0.6)	.03
Non–access site	25 (0.3)	22 (0.3)	35 (0.4)	25 (0.3)	26 (0.3)	.49
Cardiac tamponade	21 (0.2)	13 (0.2)	28 (0.3)	15 (0.2)	20 (0.2)	.75
Stent thrombosis	16 (0.2)	19 (0.2)	19 (0.2)	14 (0.2)	13 (0.2)	.57
Emergency surgery	8 (0.1)	5 (0.1)	8 (0.1)	6 (0.1)	8 (0.1)	.75

Abbreviation: LM PCI, left main percutaneous coronary intervention.

^a^ Cochran-Armitage test for trend.

### Adjusted Trends in Clinical Outcomes

After adjustment for baseline characteristics, the difference in the in-hospital mortality rate between 2019 and 2023 was attenuated (1.6% in 2019 vs 1.8% in 2023; AOR, 1.07; 95% CI, 0.85-1.35) (eFigure 1 and eTable 3 in Supplement 1). Further adjustment for procedural characteristics did not impact the changes in adjusted mortality over time (AOR, 1.08; 95% CI, 0.85-1.37) (Table 3). Similarly, no significant differences were observed for other intermediate years (2020-2022) when compared with 2019 (Table 3). In the fully adjusted model, factors associated with higher in-hospital mortality included the use of MCS, prior history of heart failure, chronic obstructive pulmonary disease, acute heart failure within 24 hours, dialysis, peripheral artery disease, chronic coronary disease, and older age (eFigure 2 in Supplement 1). In contrast, hypertension, use of atherectomy device, dyslipidemia, transradial intervention, non-ACS presentation, and elective procedures were associated with lower in-hospital mortality. These results did not significantly differ between the ACS and non-ACS groups (*P* = .75 for interaction).

**Table 3.** AORs for Clinical Outcomes After Unprotected LM PCI by Calendar Year

Year	In-hospital mortality		Composite of in-hospital mortality and procedural complication^a^	
	AOR (95% CI)^b^	*P* value	AOR (95% CI)^b^	*P* value
2020 vs 2019	0.94 (0.74-1.20)	.64	0.94 (0.79-1.11)	.46
2021 vs 2019	0.90 (0.71-1.15)	.39	1.03 (0.87-1.21)	.76
2022 vs 2019	1.12 (0.88-1.41)	.36	0.96 (0.81-1.14)	.64
2023 vs 2019	1.08 (0.85-1.37)	.51	1.08 (0.91-1.27)	.38

Abbreviations: AOR, adjusted odds ratio; LM PCI, left main percutaneous coronary intervention.

^a^ Procedural complications include cardiogenic shock or acute heart failure requiring mechanical support or inotropes, bleeding event requiring blood transfusion, cardiac tamponade, stent thrombosis based on the Academic Research Consortium definition, and emergency surgery.

^b^ Models adjustments are described in the Statistical Analysis section.

For the secondary outcome, adjusted comparisons between 2019 and 2023 showed no significant difference in the composite outcome of in-hospital mortality and procedural complications (AOR for 2023 vs 2019: 1.08; 95% CI, 0.91-1.27) (Table 3 and eTable 3 in Supplement 1). Associations between other covariates and the composite outcome are shown in eFigure 3 in Supplement 1.

## Discussion

While LM coronary artery disease has traditionally been managed surgically, current clinical practice guidelines support unprotected LM PCI as an alternative to CABG in patients with low anatomic complexity. To understand contemporary trends in the use of unprotected LM PCI and outcomes in routine clinical practice, we used data from the nationwide J-PCI registry and found no substantial increase in the use of unprotected LM PCI, which accounted for approximately 5% of all PCI procedures throughout the study period. Importantly, patients undergoing LM PCI were older and had more comorbidities over time. Although unadjusted in-hospital mortality and procedural complication rates increased modestly over time, these trends were no longer significant after adjusting for patient characteristics. Additional adjustment for procedural factors—including the increased use of radial access and mechanical circulatory support—did not meaningfully change the results. These findings indicate that the modest rise in adverse outcomes was largely explained by increasing patient complexity and that the overall safety profile of LM PCI has remained stable over time, even as it has been increasingly used in patients with higher cardiovascular risk.

This study extends the findings of prior research examining temporal trends in unprotected LM PCI in the US and UK^7,8^ While both studies reported an increase in procedure volume over time—from 0.7% to 1.3% in the US between 2009 and 2016^7^ and from 1.8% to 3.4% in the UK between 2009 and 2017^8^—recent trends over the past decade remain less well documented, and few studies have evaluated the extent to which advances in PCI techniques may have influenced outcomes. In the present study, the proportion of unprotected LM PCI cases remained stable but was higher than that of other countries,^7,8^ likely reflecting a broader application beyond CABG and increasing procedural expertise following updated clinical practice guidelines. Notably, the modest decline in the absolute number of LM PCI procedures observed over time appears to parallel a nationwide reduction in overall PCI volume in Japan, as previously reported.^22^ These system-level trends in coronary revascularization, rather than changes specific to LM disease, likely explain the observed decline in absolute LM PCI case numbers while preserving stable relative use. Regarding clinical outcomes, in-hospital mortality in the UK ranged from 1.7% to 2.2%, with no significant trend over time after adjustment^8^—consistent with our findings. In contrast, in-hospital mortality in the US was reported to be as high as 5.0%, likely reflecting selective use of LM PCI in patients with high surgical risk or multiple comorbidities.^7^ These contextual differences underscore the importance of considering patient selection and treatment intent when interpreting outcome trends across regions.

In our multivariable analysis, several procedural and patient factors were associated with in-hospital mortality. Transradial access was independently associated with better outcomes, consistent with a prior study demonstrating its benefits in reducing not only procedural complications but also major adverse cardiovascular events.^23^ Conversely, the use of MCS devices was associated with higher in-hospital mortality.^24^ In this study’s observational registry, it was difficult to define whether MCS is a marker or mediator of increased mortality. Several prior observational studies have suggested an independent association of MCS with adverse outcomes, including bleeding, acute kidney injury, cost, and mortality.^10,25^ Conversely, a recent randomized trial in a selected population suggested a protective effect on mortality,^26^ although most of the patients reported in the current study would not have met the inclusion criteria for that trial. Of interest, despite the increasing adoption of transradial access over time, access-site bleeding events slightly increased. This paradoxical finding may be partly explained by the concomitant rise in MCS use, which often requires large-bore femoral access and may offset the bleeding reduction typically seen with radial access. Future research is needed to better understand the risks and benefits of MCS in LM PCI.

Our findings offer valuable insights into the contemporary practice of unprotected LM PCI in Japan. The stability of risk-adjusted in-hospital outcomes across a large, nationwide sample suggests sustained procedural safety despite increasing case complexity. However, the absence of further improvement in clinical outcomes over time may signal a plateau in procedural gains. Notably, prior analyses from the same registry have demonstrated a volume-outcome association in LM PCI, with higher institutional procedural volume associated with lower in-hospital mortality.^21^ Given the modest decline in LM PCI case volume observed over the study period, it is possible that reduced procedural exposure at the institutional or operator level has limited opportunities for further improvement in outcomes. Periodic, high-quality reporting of procedural trends and outcomes can help identify such potential gaps in care and support iterative improvements in clinical practice. As treatment strategies and technologies continue to evolve, such benchmarking efforts will be crucial in ensuring that patients undergoing LM PCI receive optimal, evidence-based care.

### Limitations

Our findings should be interpreted in the context of several potential limitations. First, as an observational registry analysis, residual confounding cannot be fully excluded despite comprehensive multivariable adjustment. Second, detailed measures of anatomic complexity and procedural guidance, such as the SYNTAX score, specific LM lesion location or stenting strategy, reference vessel size, chronic total occlusions in non-LM territories, and intracoronary imaging guidance, were not available in the J-PCI registry. This registry was designed primarily as a nationwide quality improvement platform to monitor PCI utilization and short-term outcomes rather than to capture granular anatomic or imaging-related variables that directly inform revascularization strategy or appropriateness.^14,15^ Although we adjusted for the number of treated vessels as a crude surrogate of disease extent, this does not fully reflect the anatomic complexity that is central to guideline-recommended decision-making for LM coronary artery disease. In addition, intracoronary imaging was not recorded, precluding assessment of its temporal trends or the ability to adjust for intracoronary imaging findings. However, prior multicenter studies from Japan have shown that intravascular ultrasonography is used in a large majority of PCI procedures and that its adoption has plateaued in recent years,^20^ suggesting that temporal changes in intracoronary imaging use are unlikely to have influenced outcome trends in this cohort. Nevertheless, because intracoronary imaging use was not directly measured, residual confounding related to procedural guidance cannot be excluded and should be considered when interpreting our findings.

A third limitation is that generalizability to other health care systems and populations may be limited, because this analysis was based on data from the J-PCI registry. Fourth, because the J-PCI registry was designed to capture PCI procedures and short-term outcomes for quality improvement purposes, it does not include patients referred to CABG. Consequently, the reasons for selecting PCI over CABG were not captured, and we cannot assess whether treatment selection changed over time, which may introduce unmeasured selection bias in the observed outcome trends. Fifth, the study period included the COVID-19 pandemic, during which overall PCI volume in Japan declined, particularly in 2020, while severe presentations—including ST-segment elevation myocardial infarction, cardiogenic shock, cardiopulmonary arrest, and acute heart failure—increased, as previously reported.^27^ A similar reduction in LM PCI volume was observed in our cohort; however, the proportion of LM PCI among all PCI remained stable. Because we excluded patients with life-threatening conditions, the patient groups most affected by pandemic-related shifts were not part of the analytic population. Although our analyses adjusted for major clinical characteristics, residual pandemic-related influences on patient selection and practice patterns cannot be excluded. Sixth, the registry only captures in-hospital outcomes, and longer-term end points such as postdischarge mortality, rehospitalization, and repeat revascularization were not available. Nonetheless, the large, nationwide scope of the registry offers valuable insights into clinical trends and short-term outcomes of unprotected LM PCI in Japan.

## Conclusions

Within the national PCI registry in Japan, unprotected LM PCI consistently accounted for approximately 5% of all PCI procedures between 2019 and 2023. Over this period, the procedure was performed in increasingly older patients with more comorbidities, reflecting greater clinical complexity. Although unadjusted in-hospital mortality showed a modest increase, the findings indicate this trend was largely explained by changes in patient risk profiles, with risk-adjusted outcomes remaining stable. These findings suggest that LM PCI has been progressively adopted in higher-risk patients while maintaining consistent short-term safety, supporting its continued role as a viable revascularization strategy in contemporary practice.

## Notes

Supplement 1. **eFigure 1.** Crude and Adjusted In-Hospital Mortality in Unprotected LM PCI **eFigure 2.** Adjusted Trends in In-hospital Mortality After Unprotected LM PCI **eFigure 3.** Adjusted Trends in the Composite Outcome After Unprotected LM PCI **eTable 1.** Number of Institutions and Annual Cases Over the Study Period **eTable 2.** Trends in Clinical Outcomes After Unprotected LM PCI Among Patients Presenting ACS and Non-ACS **eTable 3.** Adjusted Odds Ratios for Clinical Outcomes After Unprotected LM PCI by Calendar Year

Supplement 2.
Data Sharing Statement

## References

[1] Vrints C, Andreotti F, Koskinas KC, ; ESC Scientific Document Group. 2024 ESC guidelines for the management of chronic coronary syndromes. Eur Heart J. 2024;45(36):3415-3537. doi:10.1093/eurheartj/ehae177 39210710

[2] Lawton JS, Tamis-Holland JE, Bangalore S, . 2021 ACC/AHA/SCAI Guideline for Coronary Artery Revascularization: a report of the American College of Cardiology/American Heart Association Joint Committee on Clinical Practice Guidelines. Circulation. 2022;145(3):e18-e114. doi:10.1161/CIR.0000000000001038 34882435

[3] McNulty EJ, Ng W, Spertus JA, . Surgical candidacy and selection biases in nonemergent left main stenting: implications for observational studies. JACC Cardiovasc Interv. 2011;4(9):1020-1027. doi:10.1016/j.jcin.2011.06.010 21939943

[4] Stone GW, Sabik JF, Serruys PW, ; EXCEL Trial Investigators. Everolimus-eluting stents or bypass surgery for left main coronary artery disease. N Engl J Med. 2016;375(23):2223-2235. doi:10.1056/NEJMoa1610227 27797291

[5] Giacoppo D, Colleran R, Cassese S, . Percutaneous coronary intervention vs coronary artery bypass grafting in patients with left main coronary artery stenosis: a systematic review and meta-analysis. JAMA Cardiol. 2017;2(10):1079-1088. doi:10.1001/jamacardio.2017.2895 28903139 PMC5710445

[6] Nakamura M, Yaku H, Ako J, ; Japanese Circulation Society Joint Working Group. JCS/JSCVS 2018 guideline on revascularization of stable coronary artery disease. Circ J. 2022;86(3):477-588. doi:10.1253/circj.CJ-20-1282 35095031

[7] Valle JA, Tamez H, Abbott JD, . Contemporary use and trends in unprotected left main coronary artery percutaneous coronary intervention in the United States: an analysis of the National Cardiovascular Data Registry Research to Practice Initiative. JAMA Cardiol. 2019;4(2):100-109. doi:10.1001/jamacardio.2018.4376 30601910 PMC6439629

[8] Kinnaird T, Gallagher S, Farooq V, . Temporal trends in in-hospital outcomes following unprotected left-main percutaneous coronary intervention: an analysis of 14 522 cases from British Cardiovascular Intervention Society database 2009 to 2017. Circ Cardiovasc Interv. 2023;16(1):e012350. doi:10.1161/CIRCINTERVENTIONS.122.012350 36649390

[9] Doll JA, Beaver K, Naranjo D, . Trends in arterial access site selection and bleeding outcomes following coronary procedures, 2011-2018. Circ Cardiovasc Qual Outcomes. 2022;15(5):e008359. doi:10.1161/CIRCOUTCOMES.121.008359 35272504

[10] Dhruva SS, Ross JS, Mortazavi BJ, . Use of mechanical circulatory support devices among patients with acute myocardial infarction complicated by cardiogenic shock. JAMA Netw Open. 2021;4(2):e2037748. doi:10.1001/jamanetworkopen.2020.37748 33616664 PMC7900859

[11] Truesdell AG, Alasnag MA, Kaul P, ; ACC Interventional Council. Intravascular imaging during percutaneous coronary intervention: JACC state-of-the-art review. J Am Coll Cardiol. 2023;81(6):590-605. doi:10.1016/j.jacc.2022.11.045 36754518

[12] Kohsaka S, Ejiri K, Takagi H, . Diagnostic and therapeutic strategies for stable coronary artery disease following the ISCHEMIA trial. JACC Asia. 2023;3(1):15-30. doi:10.1016/j.jacasi.2022.10.013 36873769 PMC9982228

[13] Maron DJ, Hochman JS, Reynolds HR, ; ISCHEMIA Research Group. Initial invasive or conservative strategy for stable coronary disease. N Engl J Med. 2020;382(15):1395-1407. doi:10.1056/NEJMoa1915922 32227755 PMC7263833

[14] Sawano M, Yamaji K, Kohsaka S, . Contemporary use and trends in percutaneous coronary intervention in Japan: an outline of the J-PCI registry. Cardiovasc Interv Ther. 2020;35(3):218-226. doi:10.1007/s12928-020-00669-z 32440831 PMC7295726

[15] Ando H, Yamaji K, Kohsaka S, ; J-PCI Registry Investigators. Japanese nationwide PCI (J-PCI) Registry annual report 2019: patient demographics and in-hospital outcomes. Cardiovasc Interv Ther. 2022;37(2):243-247. doi:10.1007/s12928-021-00832-0 35020153 PMC8753025

[16] World Medical Association. World Medical Association Declaration of Helsinki: ethical principles for medical research involving human subjects. JAMA. 2013;310(20):2191-2194. doi:10.1001/jama.2013.281053 24141714

[17] Vandenbroucke JP, von Elm E, Altman DG, ; STROBE initiative. Strengthening the Reporting of Observational Studies in Epidemiology (STROBE): explanation and elaboration. Ann Intern Med. 2007;147(8):W163-94. doi:10.7326/0003-4819-147-8-200710160-00010-w1 17938389

[18] Inohara T, Kohsaka S, Spertus JA, . Comparative trends in percutaneous coronary intervention in Japan and the United States, 2013 to 2017. J Am Coll Cardiol. 2020;76(11):1328-1340. doi:10.1016/j.jacc.2020.07.037 32912447

[19] Hicks KA, Mahaffey KW, Mehran R, ; Standardized Data Collection for Cardiovascular Trials Initiative (SCTI). 2017 Cardiovascular and stroke endpoint definitions for clinical trials. Circulation. 2018;137(9):961-972. doi:10.1161/CIRCULATIONAHA.117.033502 29483172

[20] Kuno T, Numasawa Y, Sawano M, . Real-world use of intravascular ultrasound in Japan: a report from contemporary multicenter PCI registry. Heart Vessels. 2019;34(11):1728-1739. doi:10.1007/s00380-019-01427-9 31129872

[21] Aikawa T, Yamaji K, Nagai T, . Procedural volume and outcomes after percutaneous coronary intervention for unprotected left main coronary artery disease—report from the National Clinical Data (J-PCI Registry). J Am Heart Assoc. 2020;9(9):e015404. doi:10.1161/JAHA.119.015404 32347146 PMC7428587

[22] Kohsaka S, Kumamaru H, Sawano M, . Nationwide trends in coronary revascularization in Japan, 2017 to 2023: from decline to plateau. J Am Coll Cardiol. 2025;86(23):2391-2394. doi:10.1016/j.jacc.2025.09.1594 41338846

[23] Valgimigli M, Frigoli E, Leonardi S, ; MATRIX Investigators. Radial versus femoral access and bivalirudin versus unfractionated heparin in invasively managed patients with acute coronary syndrome (MATRIX): final 1-year results of a multicentre, randomised controlled trial. Lancet. 2018;392(10150):835-848. doi:10.1016/S0140-6736(18)31714-8 30153988

[24] Zeitouni M, Marquis-Gravel G, Smilowitz NR, . Prophylactic mechanical circulatory support use in elective percutaneous coronary intervention for patients with stable coronary artery disease. Circ Cardiovasc Interv. 2022;15(5):e011534. doi:10.1161/CIRCINTERVENTIONS.121.011534 35580202

[25] Amin AP, Spertus JA, Curtis JP, . The evolving landscape of Impella use in the United States among patients undergoing percutaneous coronary intervention with mechanical circulatory support. Circulation. 2020;141(4):273-284. doi:10.1161/CIRCULATIONAHA.119.044007 31735078

[26] Møller JE, Engstrøm T, Jensen LO, ; DanGer Shock Investigators. Microaxial flow pump or standard care in infarct-related cardiogenic shock. N Engl J Med. 2024;390(15):1382-1393. doi:10.1056/NEJMoa2312572 38587239

[27] Yamaji K, Kohsaka S, Inohara T, ; J-PCI Registry Investigators. Percutaneous coronary intervention during the COVID-19 pandemic in Japan: insights from the nationwide registration data. Lancet Reg Health West Pac. 2022;22:100434. doi:10.1016/j.lanwpc.2022.100434 35330940 PMC8939342

