# Supplementary material for: Trends in Unprotected Left Main Percutaneous Coronary Intervention and Clinical Outcomes
Source: JAMA Netw Open. 2026 Feb 23;9(2):e2560422. doi: 10.1001/jamanetworkopen.2025.60422 (PMC12931462; doi:10.1001/jamanetworkopen.2025.60422)
Supplement: Supplement 1. — eFigure 1. Crude and Adjusted In-Hospital Mortality in Unprotected LM PCI eFigure 2. Adjusted Trends in In-hospital Mortality After Unprotected LM PCI eFigure 3. Adjusted Trends in the Composite Outcome After Unprotected LM PCI eTable 1. Number of Institutions and Annual Cases Over the Study Period eTable 2. Trends in Clinical Outcomes After Unprotected LM PCI Among Patients Presenting ACS and Non-ACS eTable 3. Adjusted Odds Ratios for Clinical Outcomes After Unprotected LM PCI by Calendar Year [file jamanetwopen-e2560422-s001.pdf]

Supplementary Online Content

Ikemura N, Mori Y, Chan PS, et al. Trends in unprotected left main percutaneous coronary intervention and clinical outcomes. *JAMA Netw Open*. 2026;9(2):e2560422. doi:10.1001/jamanetworkopen.2025.60422

eFigure 1. Crude and Adjusted In-Hospital Mortality in Unprotected LM PCI

eFigure 2. Adjusted Trends in In-hospital Mortality After Unprotected LM PCI

eFigure 3. Adjusted Trends in the Composite Outcome After Unprotected LM PCI

eTable 1. Number of Institutions and Annual Cases Over the Study Period

eTable 2. Trends in Clinical Outcomes After Unprotected LM PCI Among Patients Presenting ACS and Non-ACS

eTable 3. Adjusted Odds Ratios for Clinical Outcomes After Unprotected LM PCI by Calendar Year

This supplementary material has been provided by the authors to give readers additional information about their work.

© 2026 Ikemura N et al. *JAMA Network Open*.

eFigure 1. Crude and Adjusted In-Hospital Mortality in Unprotected LM PCI

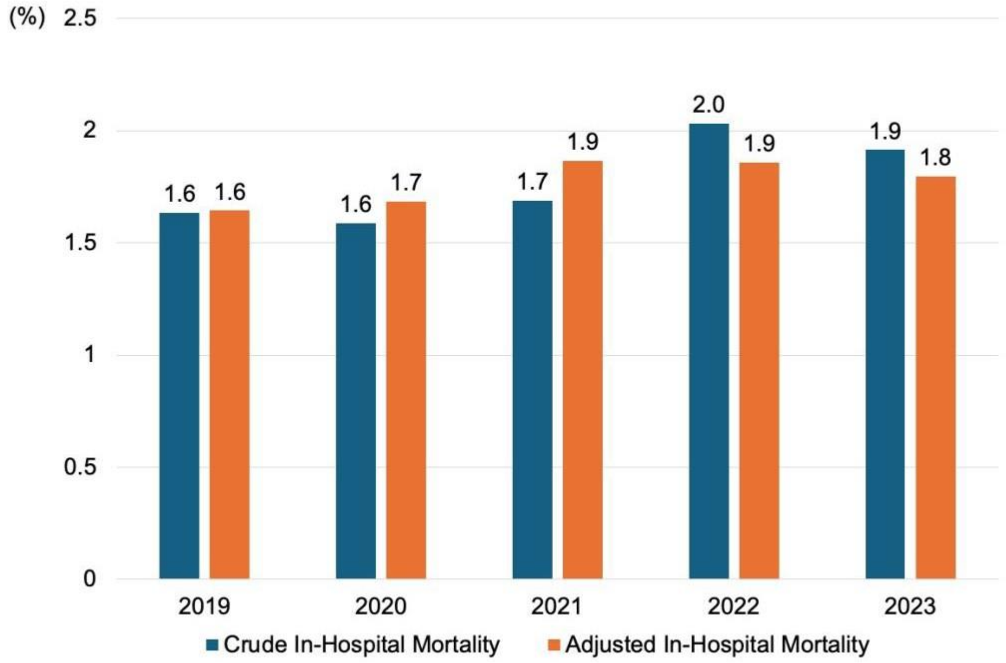

© 2026 Ikemura N et al. *JAMA Network Open*.

eFigure 2. Adjusted Trends in In-hospital Mortality After Unprotected LM PCI

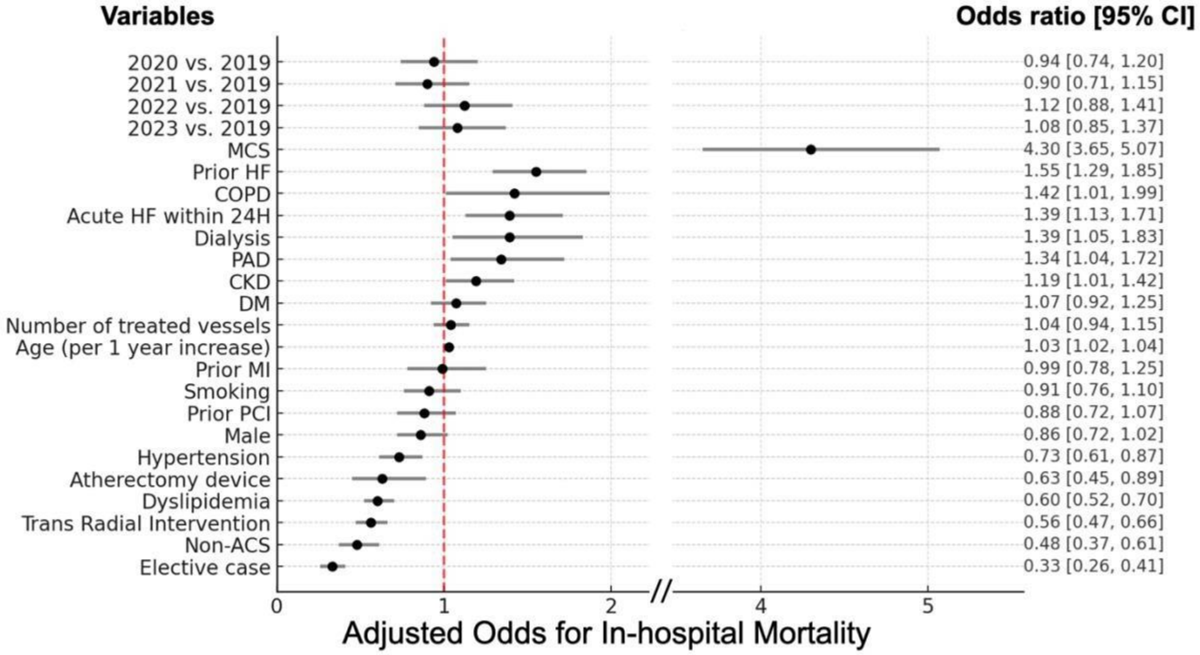

Adjustments were made in all variables shown.

Dependent variable: In-hospital mortality.

Abbreviations: MCS, mechanical circulatory support; HF, heart failure; COPD, chronic obstructive pulmonary disease; PAD, peripheral artery disease; CKD, chronic kidney disease; DM, diabetes mellitus; MI, myocardial infarction; PCI, percutaneous coronary intervention; and ACS, acute coronary syndrome

© 2026 Ikemura N et al. *JAMA Network Open*.

eFigure 3. Adjusted Trends in the Composite Outcome After Unprotected LM PCI

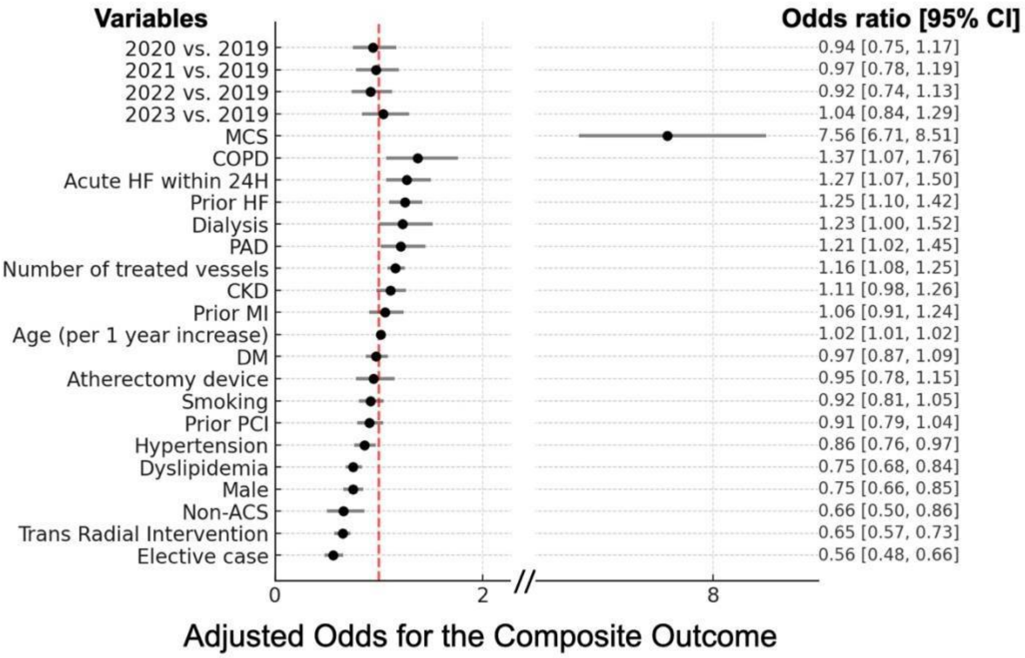

Adjustments were made in all variables shown.

Dependent variable: Composite of In-hospital mortality and procedural complication, including a cardiogenic shock or acute heart failure requiring mechanical support or inotropic, bleeding event required blood transfusion, cardiac tamponade, stent thrombosis based on the Academic Research Consortium definition, and emergency surgery.

Abbreviations: MCS, mechanical circulatory support; HF, heart failure; COPD, chronic obstructive pulmonary disease; PAD, peripheral artery disease; CKD, chronic kidney disease; DM, diabetes mellitus; MI, myocardial infarction; PCI, percutaneous coronary intervention; and ACS, acute coronary syndrome.

© 2026 Ikemura N et al. *JAMA Network Open*.

eTable 1. NUMBER of Institutions and Annual Cases Over the Study Period

	2019	2020	2021	2022	2023
Number of participating institutions	1113	1131	1150	1173	1181
Total annual cases	253,227	239,481	241,661	237,901	242,179

© 2026 Ikemura N et al. *JAMA Network Open*.

eTable 2. Trends in Clinical Outcomes After Unprotected LM PCI Among Patients Presenting ACS and Non-ACS

(A) Non-ACS

Outcomes	2019 (N=2910)	2020 (N=2737)	2021 (N=2887)	2022 (N=2860)	2023 (N=2690)	P value*
In-hospital mortality	112 (3.8)	111 (4.1)	117 (4.1)	127 (4.4)	123 (4.6)	0.12
Cardiovascular mortality	84 (2.9)	88 (3.2)	89 (3.1)	102 (3.6)	92 (3.4)	0.17
Composite outcomes of in-hospital mortality and procedural complications	204 (7)	190 (6.9)	225 (7.8)	208 (7.3)	211 (7.8)	0.20
Cardiogenic shock or acute heart failure required mechanical support or Inotropic	83 (2.9)	85 (3.1)	97 (3.4)	73 (2.6)	88 (3.3)	0.79
Bleeding event required blood transfusion Overall	27 (0.9)	23 (0.8)	42 (1.5)	29 (1.0)	33 (1.2)	0.21
-Access site	17 (0.6)	14 (0.5)	25 (0.9)	15 (0.5)	22 (0.8)	0.33
-Non-access site	10 (0.3)	9 (0.3)	17 (0.6)	14 (0.5)	11 (0.4)	0.45
Cardiac tamponade	9 (0.3)	3 (0.1)	12 (0.4)	8 (0.3)	9 (0.3)	0.51
Stent thrombosis	8 (0.3)	11 (0.4)	11 (0.4)	8 (0.3)	9 (0.3)	0.99
Emergency Surgery	4 (0.1)	4 (0.1)	4 (0.1)	1 (0.03)	3 (0.1)	0.41

(B) Non-ACS

Outcomes	2019 (N=6148)	2020 (N=5888)	2021 (N=5525)	2022 (N=5503)	2023 (N=5510)	P value*
In-hospital mortality	36 (0.6)	26 (0.4)	25 (0.5)	43 (0.8)	34 (0.6)	0.21
Cardiovascular mortality	31 (0.5)	20 (0.3)	17 (0.3)	26 (0.5)	22 (0.4)	0.73
Composite outcomes of in-hospital mortality and procedural complications	112 (1.8)	105 (1.8)	118 (2.1)	112 (2)	120 (2.2)	0.095
Cardiogenic shock or acute heart failure required mechanical support or Inotropic	51 (0.8)	50 (0.8)	63 (1.1)	51 (0.9)	59 (1.1)	0.16
Bleeding event required blood transfusion Overall	28 (0.5)	33 (0.6)	32 (0.6)	24 (0.4)	41 (0.7)	0.14
-Access site	13 (0.2)	20 (0.3)	15 (0.3)	15 (0.3)	27 (0.5)	0.036
-Non-access site	15 (0.2)	13 (0.2)	18 (0.3)	11 (0.2)	15 (0.3)	0.85
Cardiac tamponade	12 (0.2)	10 (0.2)	16 (0.3)	7 (0.1)	11 (0.2)	0.87
Stent thrombosis	8 (0.1)	8 (0.1)	8 (0.1)	6 (0.1)	4 (0.1)	0.33
Emergency Surgery	4 (0.1)	1 (0.02)	4 (0.1)	5 (0.1)	5 (0.1)	0.25

* Cochran-Armitage test for trend

© 2026 Ikemura N et al. *JAMA Network Open*.

eTable 3. Adjusted Odds Ratios for Clinical Outcomes After Unprotected LM PCI by Calendar Year

(A) In-Hospital Mortality

	Adjusted for baseline characteristics only*		Full Model**	
	Odds Ratio (95% CI)	P Value	Odds Ratio (95% CI)	P Value
2020 vs. 2019	0.94 (0.74–1.19)	0.61	0.94 (0.74–1.20)	0.64
2021 vs.2019	0.95 (0.75–1.21)	0.68	0.90 (0.71–1.15)	0.39
2022 vs. 2019	1.11 (0.89–1.40)	0.35	1.12 (0.88–1.41)	0.36
2023 vs. 2019	1.07 (0.85–1.35)	0.58	1.08 (0.85–1.37)	0.51

(B) Composite of In-hospital Mortality and Procedural Complication

	Adjusted for baseline characteristics only*		Full Model**	
	Odds Ratio (95% CI)	P Value	Odds Ratio (95% CI)	P Value
2020 vs. 2019	0.96 (0.81–1.13)	0.62	0.94 (0.79–1.11)	0.46
2021 vs.2019	1.11 (0.94–1.30)	0.19	1.03 (0.87–1.21)	0.76
2022 vs. 2019	1.01 (0.86–1.19)	0.86	0.96 (0.81–1.14)	0.64
2023 vs. 2019	1.09 (0.93–1.29)	0.26	1.08 (0.91–1.27)	0.38

***Adjustment for baseline characteristics:** patient age (per one-year increase), sex, presentation (ACS vs. non-ACS), PCI status (elective vs. non-elective), smoking, diabetes, hypertension, dyslipidemia, peripheral artery disease, chronic kidney disease (e.g., estimated glomerular filtration rate < 60 ml/min/1.73m2), dialysis, chronic obstructive lung disease, prior history of PCI, myocardial infarction, heart failure, and a history of acute heart failure with 24 hours.
****Additional adjustment for procedural characteristics:** access site (trans-radial vs. others), use of the mechanical circulatory device during the procedure, atherectomy device and a number of treated vessels.

Procedural complication includes a cardiogenic shock or acute heart failure requiring mechanical support or inotropic, bleeding event required blood transfusion, cardiac tamponade, stent thrombosis based on the Academic Research Consortium definition, and emergency surgery.

© 2026 Ikemura N et al. *JAMA Network Open*.
