# Supplementary material for: Trends in Unprotected Left Main Percutaneous Coronary Intervention and Clinical Outcomes
Source: JAMA Netw Open. 2026 Feb 23;9(2):e2560422. doi: 10.1001/jamanetworkopen.2025.60422 (PMC12931462; doi:10.1001/jamanetworkopen.2025.60422)
Supplement: Supplement 2. — Data Sharing Statement [file jamanetwopen-e2560422-s002.pdf]

Data Sharing Statement

Ikemura. Trends in Unprotected Left Main Percutaneous Coronary Intervention and Clinical Outcomes. *JAMA Netw Open*. Published February 23, 2026.
doi:10.1001/jamanetworkopen.2025.60422

Data
Data available: No
